# Genomic and functional profiles of two Antarctic chitin-degrading *Arthrobacter* strains

**DOI:** 10.1007/s00792-026-01424-7

**Published:** 2026-04-23

**Authors:** Yesenia M. Santa-Cruz Vasquez, Luis Gabriel Cueva-Yesquen, Luiz Henrique Rosa, Alysson Wagner Fernandes Duarte, Valéria Maia de Oliveira

**Affiliations:** 1 Division of Microbial Resources, Research Center for Chemistry, Biology and Agriculture (CPQBA), University of Campinas (UNICAMP), Paulínia, SP Brazil; 2 Institute of Biology, Campinas State University (UNICAMP), Campinas, SP Brazil; 3 Federal University of Minas Gerais, Belo Horizonte, Minas Gerais Brazil; 4 Federal University of Alagoas, Campus Arapiraca, Arapiraca, Brazil

**Keywords:** Antarctica, Bacterial genome, Chitinase, Enzyme prospecting

## Abstract

Chitin is the second most abundant polysaccharide in nature and constitutes a key structural component in the cell wall of fungi and invertebrate exoskeletons. Chitinases produced by Antarctic organisms have demonstrated high catalytic efficiency, being promising tools for sustainable biotechnological processes. Therefore, the objective of this study was to identify the genes responsible for chitin degradation in the genomes of two Antarctic *Arthrobacter* strains, as well as to evaluate their biotechnological potential. Whole-genome sequencing followed by phylogenomic analysis revealed that both strains 492 and 285 belong to the species *Arthrobacter psychrochitiniphilus.* Genome assembly and annotation confirmed the genetic potential for chitinase production, showing that both bacteria possess the gene encoding type C chitinase (Chi C) from the GH18 family. Genomic comparative analysis revealed that they possess a total of eleven genes related to glycoside hydrolases (GH) and carbohydrate-binding modules (CBM), highlighting GH13 and GH65. *Arthrobacter psychrochitiniphilus* 492 was classified as psychrophilic, while *A. psychrochitiniphilus* 285 was classified as mesophilic-psychrotolerant. The enzyme production by *A. psychrochitiniphilus* 285 was 19.9 U/L, in 96 h at 15 °C, and the chitinase showed activity against the *Aspergillus* sp. series *nigri* CBMAI 1846, indicating promising potential for the formulation of bioinoculants and sustainable agriculture.

**Supplementary Information:**

The online version contains supplementary material available at 10.1007/s00792-026-01424-7.

## Introduction

Chitin is the second most abundant polysaccharide in nature, following cellulose, and consists of N-acetyl-D-glucosamine (GlcNAc) units joined through β-(1→4) glycosidic bonds (Mahajan et al. 2023). It is mainly found in the cell walls of fungi and some green algae, and serves as a major structural component in the shells, cuticles, and exoskeletons of organisms such as worms, mollusks, arthropods, and crustaceans (Satitsri et al. 2020, Tsurkan et al. 2021). The abundance and versatility of the global chitin market is projected to reach 2.941 billion dollars by 2027 (Shamshina et al. 2019), driving interest in its biotechnological conversion.

Chitin treatment is needed for further biotechnological applications, and it may involve chemical, physical, and biological methods (Lv et al. 2023). Biological methods have gained greater relevance, especially because they are eco-friendly and cost-effective, driving research (Kozma et al. 2022). Chitinases are enzymes capable of degrading chitin into derivatives such as chitooligosaccharides and N-acetylglucosamine (Chen et al. 2024), which have wide applications in agriculture, medicine, food, and environmental remediation (Dhole et al. 2021). Particularly, microbial chitinases, especially those derived from cold-adapted organisms, offer advantages such as high catalytic efficiency at low temperatures, which makes them promising tools for sustainable and profitable industrial processes (Duarte et al. 2018; Liu et al. 2019).

Many bacteria degrade chitin via the hydrolytic pathway, involving the action of endo- and exo-chitinases (Dang et al. 2023; Qing et al. 2024). This pathway is employed by species such as *Streptomyces variabilis*, *Paenibacillus xylanilyticus* (Kotb et al. 2023), *Chitinolyticbacter albus* (Zhang et al. 2023), *Curtobacterium* sp. CBMAI 2942 (Vásquez et al. 2024), and *Pseudomonas* sp. GWSMS-1 (Zeng et al. 2025). Another alternative pathway is the oxidation of chitin by lytic polysaccharide monooxygenases (LPMOs), which requires oxygen and reducing agents. LPMO shows affinity for crystalline chitin and has been found in bacteria of the genus *Pseudoalteromonas* (Jiang et al. 2022a, b). Although it has been less reported, this pathway emerges as a biotechnological alternative (Zhao et al. 2024).

Antarctic bacteria are particularly interesting because their enzymes are adapted to function in low and moderate temperature environments, making them valuable for industrial processes requiring catalysis at reduced temperatures (Lima et al. 2022). The bacterium *Arthrobacter psychrochitiniphilus* GP3^T^, isolated from Antarctica, has demonstrated the ability to produce chitinase under extreme cold conditions (Wang et al. 2009). The ecological adaptation of *Arthrobacter* species is likely related to their metabolic flexibility and resilience to environmental stressors. Recent studies have identified other cold-active enzymes, such as β-galactosidase (Li et al. 2025) and α-amylase (Rutkiewicz et al. 2019) in *Arthrobacter* strains, highlighting the significant biotechnological potential of psychrophilic *Arthrobacter* species as sources of enzymes that function at low temperatures.

In a previous study, a collection of 560 bacteria isolated from Antarctica was evaluated for chitinase production (Vasquez et al. 2021). Among all the isolates, *Arthrobacter psychrochitiniphilus* 492 and *Arthrobacter cryoconiti* 285 exhibited the highest chitinolytic activity in the presence of colloidal chitin. Thus, this study aimed to genomically characterize the potential of these isolates for chitin degradation. Also, taxonomic analysis using genome sequences was performed to accurately cluster these strains into *Arthrobacter* genus. Based on chitinolytic activity, both strains were evaluated for their potential use in the biocontrol of phytopathogenic fungi.

## Materials and methods

### Bacterial strains

The strains used in this study, *Arthrobacter psychrochitiniphilus* 492 and *Arthrobacter cryoconiti* 285, were earlier isolated by Silva et al. (2018) and later identified by partial 16 S rRNA gene sequencing and verified for their chitinase production capacity by Vasquez et al. (2021). These strains were cryopreserved at -80 °C and deposited under the acronyms of CBMAI 2953 and CBMAI (in the process of being deposited) in the Brazilian Collection of Environmental and Industrial Microorganisms (CBMAI), hosted by the Research Center for Chemistry, Biology, and Agriculture (CPQBA) at the University of Campinas (UNICAMP).

### Production of chitinase by *Arthrobacter* strains

Initially, cultivation conditions were optimized for chitinase production by the *Arthrobacter cryoconiti* 285 isolate, as the production parameters for *A. psychrochitiniphilus* 492 had already been established in a previous study (Vasquez et al. 2021).

### Cultivation conditions

The bacterial inoculum was adjusted to 1 × 10⁶ cells/mL and 30 mL of this suspension were added to 270 mL of culture medium (g/L: peptone 0.3, yeast extract 0.3, K₂HPO₄ 0.7, KH₂PO₄ 0.3, MgSO₄·7 H₂O 0.5) supplemented with colloidal chitin (1%) at pH 6.0. The cultures were incubated in 1 L-Erlenmeyer flasks at 15 °C under shaking at 150 rpm for 72 h.

### Chitinase activity

Chitinase activity was assessed following a method adapted from Ulhoa and Peberdy (1992), which quantifies N-acetylglucosamine (GlcNAc) through a colorimetric reaction with 3,5-dinitrosalicylic acid (DNS). A calibration curve was constructed using standard solutions of N-acetylglucosamine (GlcNAc) (Supplementary Fig. 1), with a correlation coefficient (R²) of 0.9881. A single enzymatic unit (U) was considered as the enzyme amount capable of producing 1 µmol of N-acetylglucosamine per mL per hour, based on the calculation described by Vasquez et al. 2024. The experiments were performed in triplicate.

### Determination of the optimal growth temperature

Bacterial growth was determined by counting Colony-Forming Units (CFU)/mL. Experiments were conducted in triplicate at temperatures ranging from 5 °C to 40 °C during 72-hour incubation time to determine the temperature at which bacterial growth is maximized.

### DNA extraction, genome sequencing, and phylogenomic analysis

*Arthrobacter* strains were cultivated on R2A agar for 72 h to obtain microbial biomass. Genomic DNA was extracted using the PowerMax Soil DNA Kit (Mo Bio Laboratories, Carlsbad, CA), following the manufacturer’s instructions. DNA concentration was determined using a Qubit™ 3.0 fluorometer (Invitrogen), and purity was evaluated based on A260/A280 absorbance ratios with a NanoDrop™ 1000 spectrophotometer (Thermo Fisher Scientific). DNA integrity was assessed via 1% agarose gel electrophoresis. Whole-genome sequencing was performed at the Central Laboratory of High-Performance Technologies (LaCTAD, UNICAMP, Brazil) using paired-end libraries generated with the Nextera XT kit and sequenced on the Illumina MiSeq platform.

Quality control of the raw sequencing data was conducted using FastQC v0.11.9 (Andrews 2010) and Trimmomatic v0.39 (Bolger et al. 2014), which enabled the detection and removal of adapter sequences and primers, as well as the exclusion of reads with average Phred scores below 30. Genome assembly was carried out using SPAdes v3.13 (Bushmanova et al. 2019) and assembly quality was assessed using the QUAST v5.0.2 package (Gurevich et al. 2013). Genome completeness and potential contamination were estimated using CheckM v1.1.3. This Whole Genome Shotgun project has been deposited at DDBJ/ENA/GenBank under the accession: JBPKAJ000000000-JBPKAK000000000.

Gene prediction was carried out using Prodigal v2.6.3 (Hyatt et al. 2010). Functional annotation of the predicted genes was carried out by aligning them against the KEGG (Kanehisa et al. 2017) and eggNOG (Huerta-Cepas et al. 2019) databases using the Diamond aligner v0.9.14 (Buchfink et al. 2015). In addition, the gene prediction outputs were submitted to the eggNOG-mapper v2 platform (Cantalapiedra et al. 2021) for complementary annotation.

The phylogenomic position of the sequenced strains was inferred using the PhyloPhlAn v3.0 pipeline (Asnicar et al. 2020), which performs comprehensive phylogenetic reconstruction based on whole-genome data. Reference genomes of closely related strains were obtained from the NCBI RefSeq database. A set of 400 conserved phylogenetic markers was identified using DIAMOND v0.9.21 (Buchfink et al. 2015) and aligned using MAFFT v7.487 (Katoh and Standley 2013). The resulting alignments were concatenated for phylogenetic tree construction with FastTree v2.1.11 (Price et al. 2010). The final tree was visualized and edited using the Interactive Tree of Life (iTOL) platform (Letunic and Bork 2016), accessible at http://itol.embl.de.

### Genomic potential for carbohydrate degradation

The genomes of *Arthrobacter psychrochitiniphilus* 492 and *A. cryoconiti* 285, along with those of *A. psychrochitiniphilus* GP3 and *A. psychrochitiniphilus* DSM 23,143 retrieved from the GenBank (NCBI) database (Supplementary Table 1), were analyzed to identify potential glycoside hydrolases (GH) and carbohydrate-binding modules (CBM). Genome comparative analysis of the four strains was conducted using the COGtriangles method implemented in the COGsoft software package (NCBI) and the OrthoMCL (OMCL) clustering algorithm.

### Antifungal activity

The antifungal activity of chitinase produced by the Antarctic *Arthrobacter* strains was assessed through a paper disc diffusion assay such as described by Liu et al. (2019). The acetone-precipitated fraction (1:5) was used in the antifungal assays as the enzyme extract. Sterile filter paper discs (6.0 mm diameter) were soaked in the enzyme extract for 5 min and subsequently placed at the center of Petri dishes containing sweet potato dextrose agar (PDA). Mycelial plugs (6.0 mm diameter) from the phytopathogenic *Fusarium incarnatum* CBMAI 1981 (mango pathogen), *Fusarium complex fujikuroi* CBMAI 1274 (soil isolate), *Botrytis cinerea* CBMAI 0863 (grape isolate), and *Aspergillus* sp. series *nigri* CBMAI 1846 (tomato isolate) were placed around the enzyme-treated discs. The plates were incubated at 28 °C for 7 days. Negative control was acetone, and itraconazole (10 mg/L) was used as a positive control.

## Results and discussion

### Genome sequencing, assembly and annotation of *Arthrobacter* strains

**Fig. 1:**
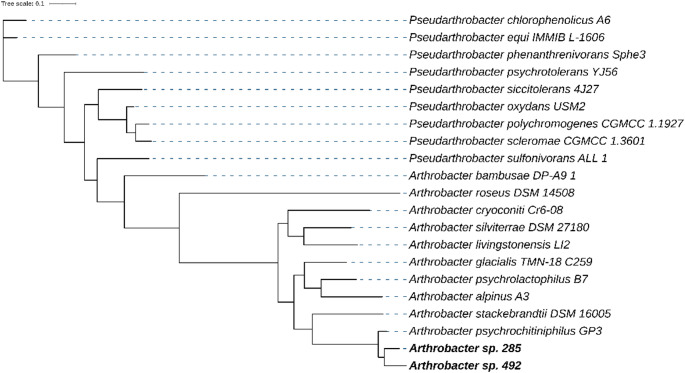
A genome-based phylogenetic tree was constructed using the genomic sequences of *Arthrobacter* strains 285 and 492. Evolutionary distances were estimated from 400 conserved proteins. Orthologous proteins were identified with DIAMOND, aligned using MAFFT, and the tree was generated with FastTree. Branch values indicate genetic distances

The de novo genome assembly enabled the reconstruction of the genomes of the Antarctic *Arthrobacter* strains. Assembly quality was evaluated based on the number of contigs, N50 value, genome completeness, and contamination levels (Supplementary Table 2). Phylogenomic analyses were performed to infer the evolutionary position of both strains with respect to other *Arthrobacter* species (Fig. 1). Also, classification at species-level was performed by calculating the OGRI (Overall Genome Related Index) values (Chun et al. 2018), including Average Nucleotide Identity (ANI) and digital DNA–DNA hybridization (dDDH) (Supplementary Table 3). Values of ANI > 96% and DDH > 75% indicate that the query strain can be taxonomically associated with the species of the reference genome (Chun et al. 2018). Strains 492 and 285 shared 98.42% and 98.15% ANI and 90.40% and 88.80% dDDH with the type strain genome of *Arthrobacter psychrochitiniphilus GP3*T (Wang et al. 2009). Thus, strain 285 was reclassified as *A. psychrochitiniphilus*.

The genus *Arthrobacter* (family Micrococcaceae, phylum Actinomycetota) has been isolated from various samples from Antarctic environments, such as soil (Contreras et al. 2025; Lopes et al. 2024; Vargas-Reyes et al. 2024), microbial mat (Lezcano et al. 2025), marine biofilm (Bisaccia et al. 2025), and other cold environments (Xu et al. 2017; Jiang et al. 2022a, b). Some species have been found in cold environments like Antarctica, including *A. psychrochitiniphilus* (Wang et al. 2009), A. *alpinus* (Zhang et al. 2010), *A. cryoconiti* (Margesin et al, 2012), and *A. ruber* (Liu et al. 2018). Their geographic distribution is primarily due to their ability to adapt to extreme environmental conditions, which allows their colonization (Dsouza et al. 2015). Additionally, studies have demonstrated their ability in producing enzymes with biotechnological applications (Kim et al. 2017; Rutkiewicz et al. 2019).

### Genomic potential for chitin degradation

Genes associated with chitin catabolism were searched in the genome from both *Arthrobacter* strains. Specifically, sequences encoding chitinase enzymes were investigated using KEGG and eggNOG databases. In the genome of *Arthrobacter psychrochitiniphilus* 492, a sequence of approximately 2,162 bp associated with the COG3325 protein (chitinase C) was detected; the same protein was also identified in *A. psychrochitiniphilus* 285. Additionally, two sequences related to the chitinase C domains in *A. psychrochitiniphilus* 285. The partial annotation of the chitinase coding genes can be attributed to limitations inherent to the sequencing method and depth and genome assembly algorithms, resulting in low genome completeness levels, and/or to database comprehensiveness. Nonetheless, the phylogenetic analysis of chitinase C of both *A. psychrochitiniphilus* strains 492 and 285 showed that these proteins present a close evolutionary relationship and share the same common ancestor.

**Fig. 2:**
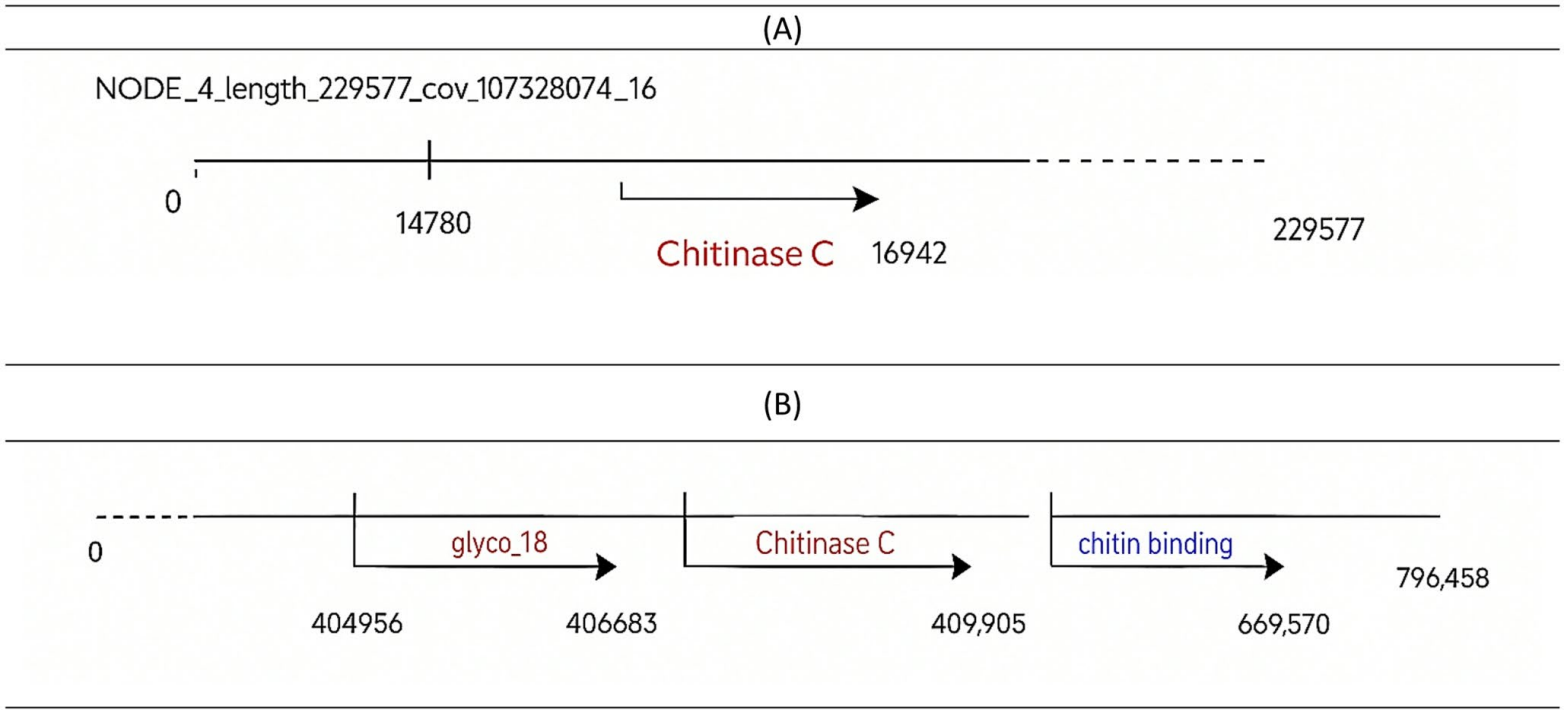
Diagram of the position of the chitinase C and chitin binding domain in the genome of the Antarctic strains. (A) *Arthrobacter psychrochitiniphilus* 492, (B)* Arthrobacter psychrochitiniphilus* 285

The functional annotation of the genome of *A. psychrochitiniphilus* 492 revealed the presence of the Chitinase C gene (*chit*C) from the GH18 family (Supplementary Table 4, Fig. 2), which is related to multiple physiological functions such as cell wall degradation, nutrition, pathogenesis, defense, and others (Chen et al. 2020). The same results were obtained for *Streptomyces diastaticus* strain CS1801 (Xu et al. 2020), a bacterium that produces extracellular chitinase. Additionally, another genomic study on *Pseudomonas* sp. TXG6-1, a Gram-negative bacterium isolated from vegetable fields in China, detected the Chitinase C gene (*PsChi*C) and insecticidal activity (Zhong et al. 2015). This gene was also found in *A. psychrochitiniphilus* 285, along with conserved domains: Glyco_18 (the domain responsible for hydrolysis) and chitin binding domain (CBD) (auxiliary domain that facilitates substrate interaction). Studies have highlighted that the presence of these domains allows for greater chitin-binding capacity and efficiency in chitinase production. In contrast, higher catalytic efficiency was reported in *Serratia marcescens*, despite having truncated chitinases, they lack the C-terminal domain (Lin et al. 2015). Genomic analysis of the Antarctic marine bacterium *Curtobacterium* sp. CBMAI 2942 showed the presence of two chitin-binding domains (ChBDs) associated with chitinaseC in its genome (Vasquez et al. 2024).

### Genomic analysis of glycoside hydrolases

**Fig. 3:**
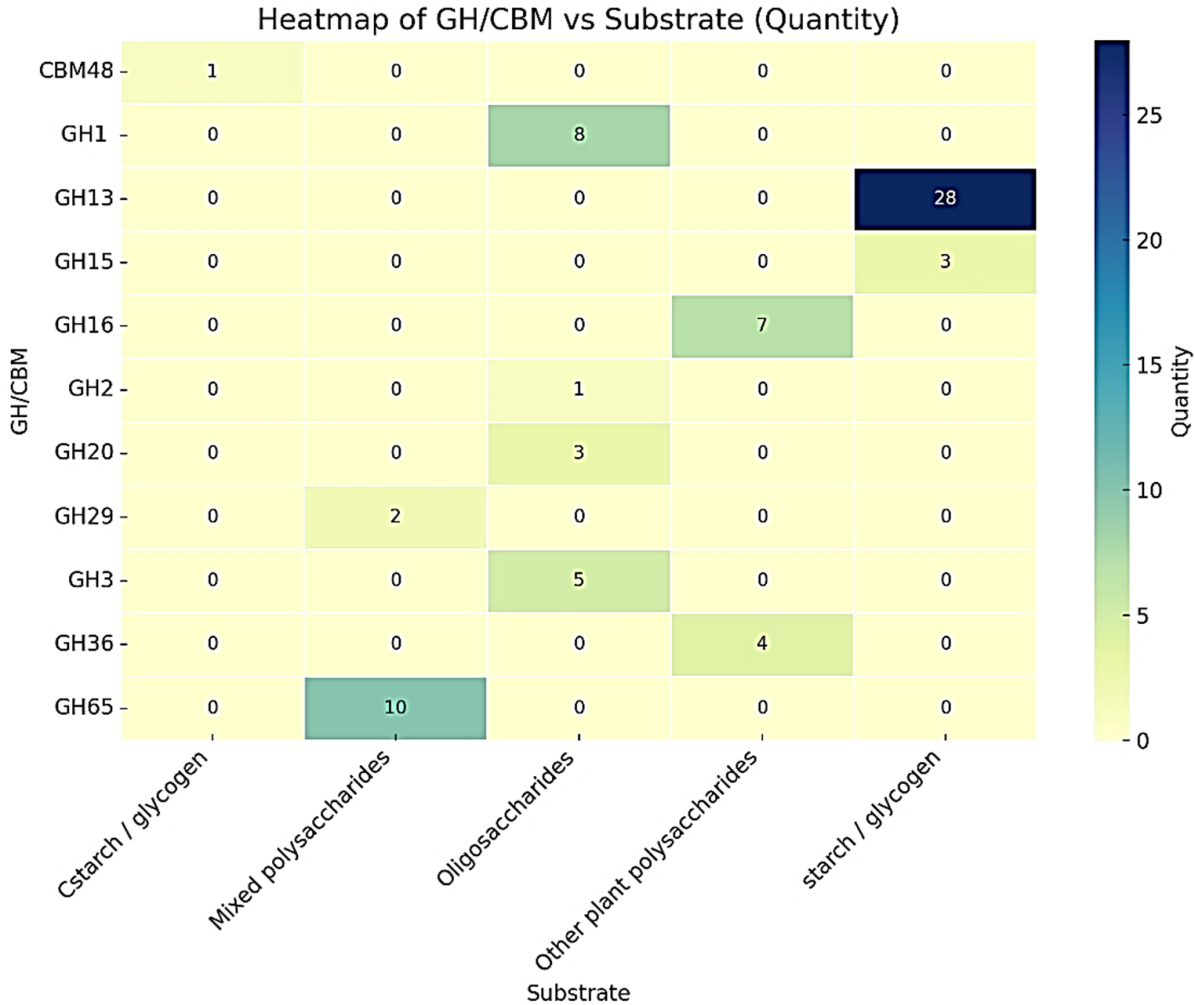
Distribution of glycoside hydrolase (GH) families and carbohydrate-binding modules (CBM) according to substrate specificity in the *Arthrobacter psychrochitiniphilus* core-genomes

Pangenomics enables the analysis of the genome (the complete set of genes) of bacteria belonging to the same species, to identify which genes are shared among strains and which are unique (Chun et al. 2017). Since the comparative evaluation involved four different genomes, the terminology “comparative evaluation of genomes” of chitinolytic bacteria of the genus Arthrobacter was chosen. A genome comparative analysis of four *Arthrobacter psychrochitiniphilus* strains, including strains 285 and 492 from this study, together with the QHLZ01 and JACBZZ01 sequences publicly available in GenBank, revealed a diverse repertoire of eleven glycoside hydrolase (GH) families, the most representative being GH13 and GH65 (Fig. 3). The genomes were analyzed to determine the presence of glycoside hydrolases (GH) and carbohydrate-binding modules (CBM) using the COGtriangles and OMCL algorithms. The pangenome obtained contained a total of 4,552 clusters, of which 2,257 clusters (49.6%) belonged to the core genome, indicating genes shared by all four strains that likely encode essential or highly conserved functions (Supplementary Table 5). Additionally, the identified GH and CBM proteins were annotated to their Pfam families, according with the CAZy database (Supplementary Table 6).

The identification of multiple hydrolytic enzymes suggests that these bacteria are adapted to survive in complex, polysaccharide-rich environments and present significant biotechnological potential that has not yet been explored. Jv and collaborators (2022) performed a pan-genomic analysis of eight strains of *Pseudoalteromonas agarivorans* (strain Hao 2018 and seven genomes available in GenBank), resulting in a total pan-genome of 5,625 genes, of which 2,331 corresponded to the core genome (41.44%), revealing a notable representation of functions associated with carbohydrate metabolism, as a source of bioactive compounds with marine biotechnological applications.

In another study, a pan-genome analysis of 40 *Bacillus paralicheniformis* strains was performed, identifying a total of 6,106 genes, with 2,282 (37.37%) to the core genome. The pan-genome revealed genes involved in carbohydrate and amino acid metabolism and secondary metabolite production (Asif et al. 2023). Studies carried out by Han et al. (2021) on the genome of *Arthrobacter* sp. PAMC25564 (Cryoconite of Wurmkogel, Austria) revealed the presence of 108 CAZymes (Carbohydrate-Active Enzymes) active at low temperatures, which have potential for further biotechnological applications.

### Determination of the optimal growth temperature in medium with colloidal chitin

**Fig. 4:**
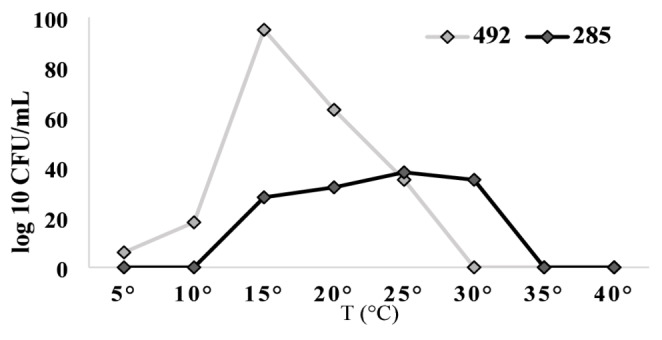
Temperature-dependent growth of *A. psychrochitiniphilus* 492 and *A. psychrochitiniphilus* 285 after 72 h of incubation at 150 rpm (5–40 °C)

The growth temperature range (5 °C to 40 °C) was assessed for both bacterial strains (Fig. 4). According to Pesciaroli et al. (2012), A. *psychrochitiniphilus* 492 was classified as psychrophilic (15 °C), while *A. psychrochitiniphilus* 285 was classified as mesophilic-psychrotolerant, with an optimal growth temperature ranging from 20 to 30 °C, the two strains belonging to *Arthrobacter psychrochitiniphilus* species exhibited different optimal growth temperatures. The optimal temperature represents one of the essential parameters for chitinase production (Reihani and Khosravi et al. 2019) and its large-scale application. Other parameters evaluated in this study were pH and agitation speed, which were similar for both strains: pH 6 and 150 rpm, respectively. Similar results were found for the psychrophilic bacterium *Pseudomonas* GWSMS-1 (Liu et al. 2019) (Fig. 4).

### Production of chitinase by *Arthrobacter psychrochitiniphilus* 285

In a previous study carried out by our group (Vasquez et al. 2021) with the *A. psychrochitiniphilus* 492 strain, a maximum chitinase production of 832.87 U/L was reached after 80 h of incubation at 15 °C, compared to its basal medium 43.4 U/L, This amount is considered high compared to other psychrophilic bacteria, such as *Sanguibacter antarcticus* KOPRI21702, which produced 90 U/L and was isolated from King George Island (Han et al. 2011), and Bacillus cereus GA6, isolated from the Himalayas, with a production of 428.57 U/mL. In contrast, strain *A. psychrochitiniphilus* 285 showed a maximum production of 19.9 U/L, without optimization, and even the application of the same optimization design as strain 492 failed to increase enzymatic activity.

This study demonstrates that strains belonging to the same species, such as *A. psychrochitiniphilus*, do not exhibit the same chitinase production potential. This factor could be related to the specific conditions required for optimal production, as demonstrated in studies with *Paenibacillus illinoisensis*, whose production was increased by immobilization in an alginate matrix (Da Silva et al. 2022). Another key factor in chitinase production is the establishment of nutritional requirements (Akram et al. 2022), which can vary among the strains studied, due to their isolation from different samples and islands in Antarctica. *A. psychrochitiniphilus* 492 showed high chitinase production at low temperatures (15 °C). Similar results were obtained with *Pseudoalteromonas* sp. DL-6 (Wang et al. 2014), *Pseudomonas* GWSMS-1, isolated (Fildes Peninsula in Antarctica) (Liu et al. 2019). These bacteria, which produce large amounts of cold-adapted chitinases, may offer several advantages in specific industrial processes, such as reduced energy consumption and substrate specificity (Siddiqui and Cavicchioli 2006), as well as various biotechnological applications in industry (Rahayu et al. 2022; Tsurkan et al. 2021).

### Antifungal activity

**Fig. 5:**
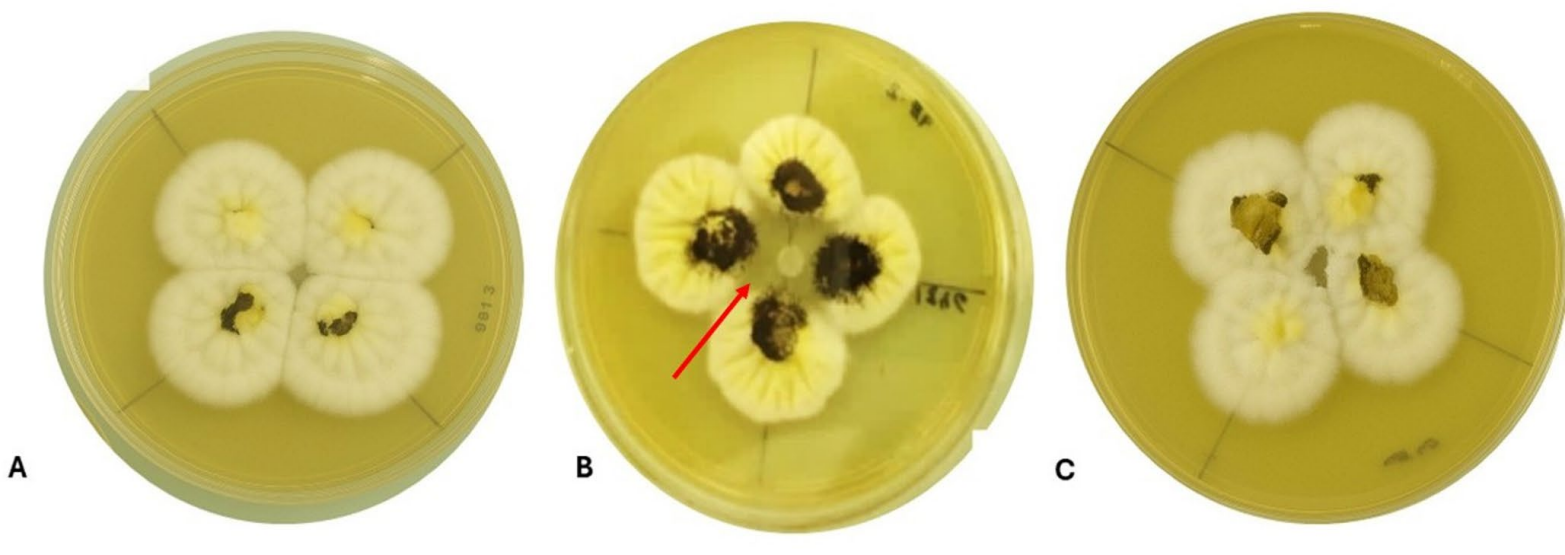
Antifungal activity with chitinase extract precipitated with acetone (1:5) against *Aspergillus* sp. series *nigri* CBMAI 1846 after 7 days of incubation. **A** *A. psychrochitiniphilus 492*; **B** *A. psychrochitiniphilus* 285; **C** acetone used as negative control

The enzymatic extract from *A. psychrochitiniphilus* 285 precipitated with acetone showed significant antifungal activity against *Aspergillus* sp. serie *nigri* CBMAI 1846 (tomato fungus), suggesting that the enzyme from this bacterium can hydrolyze the fungal cell wall, rich in chitin. Nonetheless, the potential ability of *A. psychrochitiniphilus* 285 to degrade the fungal cell wall needs to be confirmed with future assay using the purified enzyme. The extracts showed no antifungal activity against the fungi *Fusarium incarnatum* CBMAI 1981 (mango pathogen), *Fusarium complex fujikuroi* CBMAI 1274 (soil isolate), and *Botrytis cinerea* CBMAI 0863 (grape isolate). Meanwhile, the optimized extract of *A. psychrochitiniphilus* 492 did not present antifungal activity (Fig. 5, Supplementary Table 7).

Studies carried out by Vasquez et al. (2024) demonstrated the antifungal activity of chitinase produced by *Curtobacterium* sp. 458 against *Aspergillus* sp. nigri series CBMAI 1846. Another study, chitinase from *Bacillus* sp. (A9, A8, A14 and B2 strains) demonstrated antifungal activity against *Alternaria* sp. and *Colletotrichum acutatum* (Cd et al. 2021), and *Paenibacillus xylanexedens* chitinase Z2–4 demonstrated antifungal activity against the phytopathogenic fungi *Alternaria alstroemeriae*,* Botrytis cinerea*,* Sclerotinia sclerotiorum*, and *Valsa mali* (Zhang et al. 2021). In contrast, *A. psychrochitiniphilus* strains 492 did not present antifungal activity, this may be related to the fact that it does not present the Chitin binding domain (CBD) gene, which increases the affinity for the substrate and efficiency in the hydrolysis of chitin (Wang et al. 2016).

The search for new sources of biological control is justified by the need to reduce the use of synthetic agrochemicals to limit their dangerousness and bioaccumulation in the environment, with chitinase microbial with antifungal activities being related to green biotechnology. However, scaling up the production of these chitinase enzymes from *A. psychrochitiniphilus* 492 and 258 strains in bioreactor systems is necessary for larger-scale applications, as well as the purification of enzymes for a better understanding of isoforms and specific biological activity.

## Conclusions

Genomic analysis revealed the presence of genes encoding chitinase type C (Chi C) from the GH18 family related to chitinase production in the genomes of both Antarctic strains of *A. psychrochitiniphilus* (492 and 285). In addition, a total of 11 genes related to the glycoside hydrolase (GH) and carbohydrate binding module (CBM) families were identified. Results gathered in this study provided genetic evidence for the in vitro chitinolytic activity of the Antarctic strains and for the activity of the enzyme extract from *A. psychrochitiniphilus* strain 285 as an antifungal agent against *Aspergillus* sp. series *nigri* CBMAI 1846, indicating promising potential for the formulation of bioinoculants for further use in sustainable agriculture. Future studies are needed to scale up the production of chitinase by the isolate *A. psychrochitiniphilus* 285, as well as to purify the chitinases to confirm their specific antifungal activity.

## Supplementary Information

Below is the link to the electronic supplementary material.

Supplementary Material 1

## Data Availability

No datasets were generated or analysed during the current study.
