# Supplementary material for: Genomic and functional profiles of two Antarctic chitin-degrading Arthrobacter strains
Source: Extremophiles. 2026 Apr 23;30(1):16. doi: 10.1007/s00792-026-01424-7 (PMC13106269; doi:10.1007/s00792-026-01424-7)
Supplement: Supplementary file 1 — Supplementary Material 1 [file 792_2026_1424_MOESM1_ESM.docx]

**Supplementary Figure 1.** Standard curve for determination of reducing sugars by DNS using N-Acetylglucosamine (GlcNAc).

**Supplementary** **Table 1.** Data of the bacteria used for comparative analysis.

| ***Arthrobacter*** **strains** | | **Project** | **Site** | **Sequencing** | **Coverage** | **Assembly** |  |
| --- | --- | --- | --- | --- | --- | --- | --- |
| Genbank | GP3 (WGS Accession QHLZ01) | Genetic diversity of glacier-inhabiting  Cryobacterium bacteria in China | Soil samples from the Hailuogou glacier and Touming Mengke glacier | Illumina HiSeq | 250.0x | SPAdes v. 3.11 |  |
|  |  |  |  |  |  |  |  |
|  | DSM 23143 (WGS Accession JACBZZ01) | Direct Submission / Sequencing the  genomes of 1000 actinobacteria strains | NA | PacBio | 768.0x | HGAP v.smrtlink/7.0.1.66768, HGAP 4 (0.2.1) | |
| Antarctic strains | 492 | Polyphasic study of chitinase production  by Antarctic bacteria | Biofilm soil - Half Moon Island (Antarctica) | Illumina HiSeq | 250X | SPAdes v. 3.11 | |
|  | 285 |  | Sediment - King George Island (Antarctica) | Illumina HiSeq | 150 x | SPAdes v. 3.11 | |

**Supplementary Table 2.** Genome assembly statistics of the Antarctic *Arthrobacter* strains.

| **Sequence data** | **492** | **285** |
| --- | --- | --- |
| **Coverage (X)** | 250 | 150 |
| **# Contigs** | 34 | 18 |
| **Total compression** | 3463541 | 4141170 |
| **Largest *contig*** | 1767360 | 2052289 |
| **Smallest *contig*** | 1535 | 628 |
| **N50** | 1767360 | 796458 |
| **% CG** | 59.85 | 59.79 |
| **% Completeness** | 91.30 | 99.54 |
| **% Contamination** | 1.15 | 2.22 |

**Supplementary Table 3.** Estimation of ANI and dDDH values among *Arthrobacter* strains based on whole-genome sequences.

| Bacteria under study | Related bacteria | ANIb  (%) | dDDH  (%) | Diference  G:C (%) |
| --- | --- | --- | --- | --- |
| *A. psychrochitiniphilus* 492 | ***Arthrobacter psychrochitiniphilus GP3* [T]** | **98.42** | **90.40** | 0.18 |
|  | *Arthrobacter glacialis HLT2-12-2* [T] | 76.73 | 21.50 | 1.67 |
|  | *Arthrobacter alpinus DSM 22274 [T]* | 76.44 | 21.60 | 0.77 |
|  | *Arthrobacter psychrolactophilus B7* [T] | 75.94 | 21.40 | 0.81 |
| *A. cryoconiti* 285 | *Arthrobacter psychrochitiniphilus GP3* [T] | **98.15** | **88.80** | 0.12 |
|  | *Arthrobacter glacialis HLT2-12-2 [T]* | 76.32 | 21.00 | 1.73 |
|  | *Arthrobacter alpinus DSM 22274 [T]* | 76.07 | 21.00 | 0.83 |
|  | *Arthrobacter psychrolactophilus B7 [T]* | 75.76 | 20.90 | 0.87 |

**Supplementary Table 4.** Characteristics of the genes found in the genomes of the two strains of *A. psychrochitiniphilus*.

| **Strains** | **Gene ID** | **Gene name** | **Start** | **Final** | **Size (pb)** | **COG ID** | **Function COG** |
| --- | --- | --- | --- | --- | --- | --- | --- |
| *A. psychrochitiniphilus* 492 | NODE_4_length_229577_cov_107.326074_16 | Chitinase C | 14780 | 16942 | 2162 | COG3325 | Enzyme that breaks bonds in the chitin |
| *A. psychrochitiniphilus* 285 | >NODE_2_length_796458_cov_17.178526_361 | Glyco_18 | 404956 | 406683 | 1727 | COG3325 | Hydrolysis of β-1,4 bonds in chitin |
|  | NODE_2_length_796458_cov_17.178526_362 | Chitinase C | 406969 | 409605 | 2636 | COG3325 | Enzyme that breaks bonds in the chitin |
|  | NODE_2_length_796458_cov_17.178526_590 | Chitin binding domain (CBD) | 668005 | 669570 | 1565 | COG3979 | auxiliary domain that facilitates interaction with the substrate |

**Supplementary Table 5.** Comparative genomic analysis metrics.

| **Analysis metrics** | **Number_of_clusters** | **Interpretation** |
| --- | --- | --- |
| Complete pangenome | 4552 | Represents all the different genes found |
| core-genome | 2257 | Number of essential or highly conserved genes shared across the four strains |

**Supplementary Table 6**. Identified glycoside hydrolases (GH) and carbohydrate-binding modules (CBM) in the genomes of the *A. psychrochitiniphilus* strains.

| **GH/CBM** | **Pfam IDs** | **Main biological functions** | **Substrate** |
| --- | --- | --- | --- |
| GH1 | PF00232 | B-glucosidase (EC 3.2.1.21); B-galactosidase (EC 3.2.1.23); 6-phospho-B-galactosidase (EC 3.2.1.85); 6-phospho-B-glucosidase (EC 3.2.1.52); lactase-phlorizin hydrolase (EC 3.2.1.62), lactase (EC 3.2.1.108); B-mannosidase (EC 3.2.1.25); myrosinase (EC 3.2.1.147). | Oligosaccharides |
| GH2 | PF02837 | B-glucuronidase (EC 3.2.1.31) | Oligosaccharides |
| GH3 | PF00933 PF01915 | B-glucuronidase (EC 3.2.1.31); B-xylosidase (EC 3.2.1.37); N-acetyl B-glucosaminidae (EC 3.2.1.58); cellodextrinase (EC 3.2.1.74); exo-1,3-1,4-glucanase (EC 3.2.1) | Oligosaccharides |
| GH13 | PF00128 | x-amylase (EC 3.2.1.1); pullulanase (EC 3.2.1.41); cyclomaltodextrinase (EC 3.2.1.93); oligo-x-glucosidase (EC 3.2.1.10); maltogenic amylase (EC 3.2.1.133); neupullulanase (EC 3.2.1.135); X-glucosidase (EC 3.2.1.20); maltotetraoseforming x-amylase (EC 3.2.1.60); isoamylase (EC 3.2.1.68); glucodextranase (EC 3.2.1.70); maltohexaose-forming x-amylase (EC 3.2.1.98); maltotriose-forming x-amylase (EC 3.2.1.116); branching enzyme (EC 2.4.1.18); trehalose synthase (EC 5.4.99.16); 4-x-glucanotransferase (EC 2.4.1.25); maltopentaose-forming x-amylase (EC 3.2.1.-); amlylosucrase (EC 2.4.1.4); sucrose phosphorylase (EC 2.4.1.7); malto-oligosyltrehalose trehalohydrolase (EC 3.2.1.141); isomaltulose synthase (EC 5.4.99.11); malto-oligosyltransferase synthase (EC 5.4.99.15); amylo-X-1,6-glucosidase (EC 3.2.1.33), x-1,4-glucan:phosphate x-maltosyltransferase (EC 2.4.99.16); amino acid transporter; (retaining) sucrose 6(F)-phosphate phosphorylase (EC 2.4.1.139); (retaining) glucosylglycerol phosphorylase (EC 2.4.1.359); glucosylglycerate phosphorylase (EC 2.4.1.352); (retaining) sucrose x-glucosidase (EC 3.2.1.48); oligosaccharide x-4-glucosyltransferase (EC 2.4.1.161). | starch / glycogen |
| GH15 | PF00723 | glucoamylase (EC 3.2.1.3); a-glucosidase (EC 3.2.1.20);glucodextranase (EC 3.2.1.70) | starch / glycogen |
| GH16 | PF00722 | Lichenase; xyloglucan xyloglucosyltransferase; agarase; K-carrageenase; endo-,B-1,3-glucanase; endo-,B-1,3- 1,4-glucanase; endo-,B-galactosidase | Other plant polysaccharides |
| GH20 | PF 00728 PF02838 | B-hexosaminidase; lacto-N-biosidase; B-1,6-N-acetylglucosaminidase; B-6-S03-N-acetylglucosaminidase | Oligosaccharides |
| GH29 | PF01120 | X-L-fucosidase (EC 3.2.1.51); X-1,3/1,4-L-fucosidase (EC 3.2.1.111) | Mixed polysaccharides |
| GH36 | PF16874 PF16875 PF02065 | X-galactosidase (EC 3.2.1.22); x-N-acetylgalactosaminidase (EC 3.2.1.49); stachyose syntase (EC 2.4.1.67); raffinose synthase (EC 2.4.1.82) | Other plant polysaccharides |
| GH65 | PF03633 | a,a-trehalase (EC 3.2.l.28); maltose phosphorylase (EC 2.4.l.8); trehalose phosphorylase (EC 2.4.l.64); kojibiose phosphorylase (EC 2.4.l.230); trehalose-6-phosphate phosphorylase (EC 2.4.l.216); nigerose phosphorylase (EC 2.4.1.279); 3-0-X-glucopyranosyl-L-rhamnose phosphorylase (EC 2.4.l.282); 2-0-B- glucopyranosylglycerol: phosphate B-glucosyltransferase (EC 2.4.1.-); X-glucosyl-1,2-B-galactosyl-L-hydroxylysine X-glucosidase (EC 3.2.1.107); 1,3-Í±-oligoglucan phosphorylase (EC 2.4. l.334) | Mixed polysaccharides |
| CBM32 | PF00754 | Non-reducing terminus of N-acetyllactosamine-binding module | NA |
| CBM48 | PF02922 | Glycogen-binding function | starch / glycogen |
| CBM50 | PF01476 | Modules of approximately 50 amino acid residues have been identified linked to various enzymes belonging to the GH18, GH19, GH23, GH24, GH25, and GH73 families, all of which are associated with the hydrolysis of chitin or peptidoglycan. | NA |

**Supplementary Table 7.** Antifungal activity assay with chitinase extract precipitated with acetone (1:5) against phytopathogenic fungi isolates after 7 days of incubation**.** Acetone was used as negative control.

| Antifungal activity | *A. psychrochitiniphilus*  285 | *A. psychrochitiniphilus* 492 | Neg. control | Itraconazol |
| --- | --- | --- | --- | --- |
| *Botrytis cinerea* CBMAI 0863 | 0 mm | 0 mm | 0 mm | 0 mm |
| *Aspergillus* sp. series nigri CBMAI 1846 | 15 mm | 0 mm | 0 mm | 26 mm |
| *Fusarium* complex fujikuroi CBMAI 1274 | 0 mm | 0 mm | 0 mm | 0 mm |
| *Fusarium incarnatum* CBMAI 1981 | 0 mm | 0 mm | 0 mm | 0 mm |
